# The Benefits and Pitfalls of Using an Autologous Dermal Flap in Immediate Implant-Based Reconstruction

**DOI:** 10.7759/cureus.14144

**Published:** 2021-03-27

**Authors:** Natalia L Garibotto

**Affiliations:** 1 Breast and Endocrine Surgery, St Georges Hospital, Sydney, AUS

**Keywords:** autologous dermal flap, dermal sling, breast reconstruction

## Abstract

One of the challenges of implant breast reconstruction post-subcutaneous mastectomy is coverage of the inferior pole of the implant to provide a barrier between the implant and skin. Numerous biological and synthetic meshes are available on the market for this purpose; however, they are often very costly and carry all the risks of using a foreign body. In patients with large ptotic breast, the skin of the inferior mastectomy flap can be used instead. A number of techniques and variations have been developed over the last 40 years driven by the increasing cost of healthcare and acceptance of breast reconstruction as vital part of breast cancer care and survivorship. This review outline the benefits and pitfalls of using an autologous dermal flap in breast construction and the variations in published use.

## Introduction and background

The use of an autologous dermal sling (DS) created from the de-epithelialized skin of an inferior mastectomy flap is sometimes described as the Bostwick technique after it was described in a plastic surgery textbook in the 1990s [1]. This description covered an immediate implant with two layers of vascularized tissue after a ‘Wise pattern’ mastectomy with a free nipple graft. This was termed a DS. However, publications describing the use of an autologous dermal flap (ADF) have been published as early as the late 1970s [2]. A Medline search combined with reference list search was performed to describe the various published techniques in breast reconstruction using an ADF and the general benefits and pitfalls of the procedure.

## Review

Hansson et al. [3] published a systematic review on the number of techniques used and synthesized it down to nine categories (Table 1). An ADF (termed a DS in this paper) can be used in the technique above (‘classic’ DS) or with a non-Wise pattern mastectomy. It can be used in conjunction with a synthetic mesh or acellular dermal matrix (ADM), with or without an implant, or simply as a buttress for a suture line or T-junction. It can be used in a one- or two-stage reconstruction. One of the primary outcomes was to grade the level of evidence in the literature that used an ADF in breast reconstruction using the Oxford Centre for Evidence-Based Medicine 2009 guidelines. Generally, the level of evidence in most of the studies used was of low to very low quality, being mostly case and cohort studies. They could not find any randomized controlled trials for the use of any of these flaps. This is inherently difficult as there is no established standard technique for the use of ADF as a comparison. Furthermore, randomization of patients would be difficult as they are highly selected to undergo this technique in the first place. However, the main conclusion was that the ADF was a good option in patients with macromastia and significant ptosis. An ideal nipple to IMF (inframammary fold) length of 8-15 cm was suggested; however, this depends on the size of end result.

**Table 1:** Surgical modifications of ADF. ADF, autologous dermal flap; DS, dermal sling; NAC, nipple-areola complex

Modifications
1. Classic DS with minor modifications – complete implant coverage with ADF
2. Non-Wise-pattern mastectomy DS – skin is draped ‘vest over pants’ without T-junction
3. NAS-bearing DS – the NAC carries its own pedicle
4. DS in combination with a matrix/mesh
5. DS as a suture line protection technique – only a small area of de-epithelialized skin is used to buttress the suture line
6. DS with a modified circulatory basis – flap has a different vascular pedicle other than inferior i.e. superior medial
7. DS without an implant – “goldilocks mastectomy”
8. DS as an immediate-delayed technique – ADF used to maintain skin for delayed tissue reconstruction
9. Pre-pectoral DS – ADF used in pre-pectoral implant rather than subpectoral

In many circumstances, the use of an ADF negates the need for a synthetic mesh or matrix to provide implant coverage. These are often very expensive, especially the human-derived ADM. A literature review performed by a U.S. group comparing ADM in patients undergoing a single-staged immediate breast reconstruction demonstrated a cost benefit to using an ADF [4]. This cost benefit was durable, with a complication rate of up to 20%. Most studies analyzed in this paper quoted an average of 10.5% complication rate with ADF compared to 11% with an ADM. This benefit is further strengthened by the fact that most women undergoing an ADF have a higher BMI, which is an independent risk factor for complications [5]. This is because the breast needs to be sufficiently large in order to us the inferior mastectomy flap as an ADF. This study did demonstrate a reduced rate of infection, seroma, and hematoma with the use of an ADF; however, mastectomy skin necrosis and explantation were higher. An ADF was still not recommended in smokers, as smoking increases the risks of complications in all breast cosmetic and reconstructive procedures [6].

In general, however, most of the reviewed literature supported the fact that using an ADF resulted in lower complications overall and was safe and effective in select patients [7,8]. Ladizinsky et al. [1] reported an overall complication rate of 23.5%, with the highest being in the patients who smoked, had a BMI of >35, and had a direct to implant reconstruction. A further risk factor was a volume of breast excised >700 gm [9]. The use of a tissue expander to reduce the tension on the mastectomy flap correlated with a lower flap necrosis rate. The patient population that benefited the most was the BRCA group undergoing bilateral prophylactic mastectomy. They surmised that the lack of lymph node biopsy, younger age, and less co-morbidities favored a good outcome and thus may be ideal in this patient population. Other studies with smaller series published favorable results with low complication rates using an ADF [10-13]. The use of a non-autologous ADM has been associated with an increase in infection rate, especially in patients with a high BMI, which is the patient population that usually benefits the most from and ADF [5].

The T-junction of a Wise pattern mastectomy is prone to wound breakdown and loss or extrusion of implant if used to reconstruct the breast. An ADF can be used as vascularized tissue to cover the implant and provide a scaffold for epithelialization and a buffer to wound breakdown or flap necrosis particularly in the T-zone. The rate of implant exposure and extrusion was lower when an ADF was used in this circumstance [14]. Similarly, preservation of the nipple during a nipple-sparing mastectomy in a large ptotic breast has a higher risk of nipple and skin flap necrosis. This is due to the excessive length of vascular pedicle to the extremity of the breast. Lewin et al. developed a novel technique using a bipedicle dermal flap. A skin-reducing Wise pattern utilizing an inferior pedicle is marked. The inferior pedicle and usual area of skin excision is de-eplithelialized. The nipple-sparing mastectomy is performed through an incision through the lateral edge of the de-epithelialized skin. A subpectoral implant is placed, and the skin flaps are sutured over the de-epithelialized skin. The vascularity of the ADF or nipple is rarely compromised due to the wide inferior vascular pedicle [15]. Most papers describing the use of an ADF in preserving the nipple do so by a free nipple graft over de-eplithelialized skin in the upper flaps in the pre-marked position.

The disadvantage of ADF most often quoted is the limitation to the large and ptotic breast, and this is true of the classic DS as a large amount of tissue is required to cover the implant. A nipple-to-IMF distance of greater than 8 cm and a nipple-to-sternal notch greater than 25 cm are the ideal dimensions for an adequate ADF [10]. Nair et al. [16], however, used ADF in medium and small non-ptotic breast but employed an expander rather than permanent implant to achieve the desired size subverting this limitation. There was no discussion, however, about later exchange of the expander implant, which leads one to think that they used the expander as a permanent implant. Modern expanders are not designed to stay for a long term and need to be exchanged for a permanent implant. Hammond et al. [17] and Halls [18] also employed the use of ADF in a two-staged reconstruction using a tissue expander in the early 2000. They reported using the lateral end of the inframammary wound to exchange to a permanent implant at a later stage. This did not appear to compromise the ADF or the implant.

Nava et al. [10] then adopted the single-stage reconstruction with a direct to permanent implant in 2006, which is widely quoted in much of the following literature [7,19-20]. They have published further papers on this technique [21] always using a subpectoral pocket. The ADF is sutured to the lower end of the pectoralis major muscle. A prepectoral approach can be used if combined with a mesh or ADM. Caputo et al. [22] created a pocket using a porcine-derived ADM to provide superior pole coverage to an implant sutured to an inferior ADF and the pectoralis major muscle. Avoidance of dissection of the submuscular plane resulted in less postoperative pain with no animation on contraction of pectoralis major muscle while achieving a good cosmetic outcome. Implant loss due to T-junction breakdown was not experienced in this small study. This was again attributed to the coverage of the implant in the lower pole with well vascularized ADF.

The use of an ADF in patients who have had neoadjuvant or previous chemotherapy or radiotherapy has yet to be fully investigated and would be of great value with the increasing use of reverse sequencing. Autologous tissue reconstruction is usually favored if reverse sequencing is used due to the perceived high rate of capsular contractures with implant-based reconstruction. Monrigal et al. recorded a 23% capsular contracture rate in their five-year follow-up after neoadjuvant radiotherapy [23]. A recent review, however, found that the rate of complications after neoadjuvant radiotherapy is no higher than adjuvant radiotherapy even when an implant-based reconstruction is used [24]. Most were implants placed with a latissimus dorsi flap for coverage [25,26]. The use of an ADF may be beneficial in this circumstance; however, it still relies on coverage of the implant with irradiated tissue.

## Conclusions

The use of an ADF in implant-based breast reconstruction is a cost-effective, safe, and reliable method in women with a range of breast sizes. The ADF can be tailored to breast size and reconstruction method using a wide variety of techniques with less complication rates and lower cost.

## References

[1] Ladizinsky DA, Sandholm PH, Jewett ST, Shahzad F, Andrews K (2013). Breast reconstruction with the Bostwick autoderm technique. Plast Reconstr Surg.

[2] Biggs TM, Brauer RO, Wolf LE (1977). Mastopexy in conjunction with subcutaneous mastectomy. Plast Reconstr Surg.

[3] Hansson E, Jepsen C, Hallberg H (2019). Breast reconstruction with a dermal sling: a systematic review of surgical modifications. J Plast Surg Hand Surg.

[4] Krishnan NM, Chatterjee A, Van Vliet MM, Powell SG, Rosen JM, Nigriny JF (2013). A comparison of acellular dermal matrix to autologous dermal flaps in single-stage, implant-based immediate breast reconstruction: a cost-effectiveness analysis. Plast Reconstr Surg.

[5] Liu AS, Kao HK, Reish RG, Hergrueter CA, May JW Jr, Guo L (2011). Postoperative complications in prosthesis-based breast reconstruction using acellular dermal matrix. Plast Reconstr Surg.

[6] Goltsman D, Munabi NCO, Ascherman JA (2017). The association between smoking and plastic surgery outcomes in 40,465 patients: an analysis of the American College of Surgeons National Surgical Quality Improvement Program data sets. Plast Reconstr Surg.

[7] Goyal A, Wu JM, Chandran VP, Reed MW (2011). Outcome after autologous dermal sling-assisted immediate breast reconstruction. Br J Surg.

[8] Irwin GW, Black A, Refsum SE, McIntosh SA (2013). Skin-reducing mastectomy and one-stage implant reconstruction with a myodermal flap: a safe and effective technique in risk-reducing and therapeutic mastectomy. J Plast Reconstr Aesthet Surg.

[9] Inbal A, Gur E, Lemelman BT, Barsuk D, Menes T, Leshem D, Barnea Y (2017). Optimizing patient selection for direct-to-implant immediate breast reconstruction using Wise-pattern skin-reducing mastectomy in large and ptotic breasts. Aesthetic Plast Surg.

[10] Nava MB, Cortinovis U, Ottolenghi J (2006). Skin-reducing mastectomy. Plast Reconstr Surg.

[11] Peker F, Yuksel F, Karagoz H, Ozturk S (2015). Breast reconstruction using de-epithelialized dermal flap after vertical-pattern skin-sparing mastectomy in macromastia. ANZ J Surg.

[12] Ross GL (2012). One stage breast reconstruction following prophylactic mastectomy for ptotic breasts: the inferior dermal flap and implant. J Plast Reconstr Aesthet Surg.

[13] King IC, Harvey JR, Bhaskar P (2014). One-stage breast reconstruction using the inferior dermal flap, implant, and free nipple graft. Aesthetic Plast Surg.

[14] Corban J, Shash H, Safran T, Sheppard-Jones N, Fouda-Neel O (2017). A systematic review of complications associated with direct implants vs. tissue expanders following Wise pattern skin-sparing mastectomy. J Plast Reconstr Aesthet Surg.

[15] Lewin R, Jepsen C, Hallberg H, Hansson E (2018). Immediate breast reconstruction with a wise pattern mastectomy and NAC-sparing McKissock vertical bipedicle dermal flap. J Plast Reconstr Aesthet Surg.

[16] Nair A, Jaleel S, Abbott N, Buxton P, Matey P (2010). Skin-reducing mastectomy with immediate implant reconstruction as an indispensable tool in the provision of oncoplastic breast services. Ann Surg Oncol.

[17] Hammond DC, Capraro PA, Ozolins EB, Arnold JF (2002). Use of a skin-sparing reduction pattern to create a combination skin-muscle flap pocket in immediate breast reconstruction. Plast Reconstr Surg.

[18] Halls MJ (2012). Superiorly based single mastectomy flap with inferiorly based dermal flap and anchorage of the inframammary crease procedure for immediate breast reconstruction in patients with ptosis. Plast Reconstr Surg.

[19] De Vita R, Pozzi M, Zoccali G, Costantini M, Gullo P, Buccheri EM, Varanese A (2015). Skin-reducing mastectomy and immediate breast reconstruction in patients with macromastia. J Exp Clin Cancer Res.

[20] Dietz J, Lundgren P, Veeramani A (2012). Autologous inferior dermal sling (autoderm) with concomitant skin-envelope reduction mastectomy: an excellent surgical choice for women with macromastia and clinically significant ptosis. Ann Surg Oncol.

[21] Nava MB, Ottolenghi J, Pennati A (2012). Skin/nipple sparing mastectomies and implant-based breast reconstruction in patients with large and ptotic breast: oncological and reconstructive results. Breast.

[22] Caputo GG, Marchetti A, Dalla Pozza E, Vigato E, Domenici L, Cigna E, Governa M (2016). Skin-reduction breast reconstructions with prepectoral implant. Plast Reconstr Surg.

[23] Monrigal E, Dauplat J, Gimbergues P (2011). Mastectomy with immediate breast reconstruction after neoadjuvant chemotherapy and radiation therapy. A new option for patients with operable invasive breast cancer. Results of a 20 years single institution study. Eur J Surg Oncol.

[24] O' Halloran N, McVeigh T, Lowery A, Martin J, Kerin M (2017). Neoadjuvant chemo-radiation therapy and breast reconstruction - the potential for superior cosmetic outcomes in the treatment of breast cancer. British J Surg.

[25] Giacalone PL, Rathat G, Daures JP, Benos P, Azria D, Rouleau C (2010). New concept for immediate breast reconstruction for invasive cancers: feasibility, oncological safety and esthetic outcome of post-neoadjuvant therapy immediate breast reconstruction versus delayed breast reconstruction: a prospective pilot study. Breast Cancer Res Treat.

[26] Haydon N, Southwell-Keely J, Moisidis E (2014). 'Imbricated dermal flap': a novel technique for autologous augmentation in immediate breast reconstruction after skin-sparing mastectomy. Eur J Surg Oncol.

